# Comorbidities and Crash Involvement among Younger and Older Drivers

**DOI:** 10.1371/journal.pone.0094564

**Published:** 2014-04-10

**Authors:** Michela Papa, Virginia Boccardi, Raffaele Prestano, Edith Angellotti, Manuela Desiderio, Luigi Marano, Maria Rosaria Rizzo, Giuseppe Paolisso

**Affiliations:** Department of Internal Medicine, Surgical, Neurological, Metabolic Disease and Geriatric Medicine, Second University of Naples, Naples, Italy; Cardiff University, United Kingdom

## Abstract

Previous studies identified comorbidities as predictors of older driver performance and driving pattern, while the direct impact of comorbidities on road crash risk in elderly drivers is still unknown. The present study is a cross-sectional aimed at investigating the association between levels of comorbidity and crash involvement in adult and elderly drivers. 327 drivers were stratified according to age range in two groups: elderly drivers (age ≥70 years old, referred as older) and adult drivers (age <70 years old, referred as younger). Driving information was obtained through a driving questionnaire. Distance traveled was categorized into low, medium and high on the basis of kilometers driven in a year. CIRS-illness severity (IS) and CIRS-comorbidity indices (CI) in all populations were calculated. Older drivers had a significantly higher crash involvements rate (p = .045) compared with the younger group based on the number of licensed drivers. Dividing comorbidity indices into tertiles among all licensed subjects, the number of current drivers significantly decreased (p<.0001) with increasing level of comorbidity. The number of current drivers among older subjects significantly decreased with increasing comorbidity level (p = .026) while no difference among younger group was found (p = .462). Among younger drivers with increasing comorbidity level, the number of road accidents significantly increased (p = .048) and the logistic regression analysis showed that comorbidity level significantly associated with crash involvement independent of gender and driving exposure. Older subjects with high level of comorbidity are able to self-regulate driving while comorbidity burden represents a significant risk factor for crash involvements among younger drivers.

## Introduction

The older population continuously grows and the corresponding increase in older drivers creates numerous challenges to driving authorities and public safety. However, compared with younger drivers, older drivers have lower rates of crash involvements, largely because fewer older subjects keep their license for long, and those who do drive fewer miles [1], [2] Because of age-related frailty, elderly drivers are at an increased risk for crash-related injury or death compared to younger drivers [3], [4]. Previous studies indicated that functional impairments (mainly involving vision, cognition, and physical ability), comorbidities and polypharmacy, rather than age alone, contribute to the crash involvement risk and poor driving performance observed among elderly populations [5]–[10]. In fact, as people age, they are more likely to develop chronic medical conditions which are vehicle crash predictors as well. Strong evidences also show that poor health is strongly associated with driving cessation among elderly people [11]–[13].

Currently, our understanding of the effect of comorbidities is limited to epidemiological study of fatality or injury [4] and the observation that comorbidities are predictors of older driver performance and driving pattern [1], [14], while the direct impact of comorbidities on road crash risk in elderly drivers is still unknown. Some studies suggest that an improvement can be made through a reduction in driving among people with poorer health, but the association between comorbidity burden, age and vehicle crash involvement remains unexplored. There are a number of comorbidity indices that identify and summarize comorbidity burden. The Cumulative Illness Rating Scale (CIRS) is one of the few standardized instruments for the rating of medical problems by organ system [15], [16] and is able to predict outcome in a variety of conditions [17]–[21].

In light of such evidences, our study aimed at investigating and elucidating the impact of comorbidities measured by CIRS scores on road crash involvement risk among elderly and adult drivers.

## Method

### Ethics statement

This investigation has been conducted in accordance with ethical standards. After a clear explanation of the potential risk of the study, all subjects were provided with written informed consent to participate in the study, which was approved by the Ethical Committee of the Second University of Naples.

### Study population

600 unselected consecutive subjects who were referred to our Department of Internal Medicine for diagnosis and treatment of age related diseases over last two years have been screened. Exclusion criteria were: age under 40 years, neurological diseases (including Parkinson's disease and Alzheimer's disease) and psychiatric disorders (psychosis, bipolar illness and major depression). All subjects taking hypnotic and anxiolytic drugs were ruled out. No subject declined consent. We obtained usable data from 562 subjects, who had volunteered to take part. The data were collected from January to July 2013. The analysis was conducted on 327 current drivers of passenger vehicles, defined as active drivers with current driver's license. For the remainder of this paper, the term “driver” refers to passenger vehicle driver. Crash was defined as that which occurred on a public road, involved vehicle damage where participant was the driver [22]. To examine if comorbidities relate differently to crash risk among elderly and adult drivers, the population was stratified into two groups according to age range: older drivers included subjects aged 70 and older and adult drivers group were younger than 70 and older than 40. For the remainder of this paper, the term “younger” refers to adult subjects group and the term “older” refers to elderly subjects group. Data collection included an interview concerning demographics, health-related behaviors, functional status, medical conditions, and cognitive function. All subjects were asked if they have a driver's license, if they normally drive, and if they had road crashes in the last 5 years. Measure of traffic exposure was obtained by asking participants how far they would usually drive in a year by memory. Distance traveled was then categorized into low (less than 6000 km/year), medium (6000–12000 km/year), or high (greater than 12000 km/year). Research on self-reported mileage suggests that this information is accurate compared with actual mileage, even among older drivers [22], [23].

### Comorbidity assessment

Comorbidity was assessed using the Cumulative Illness Rating Scale (CIRS). This rating scale consists of 14 items covering: heart, hypertension, vascular and respiratory disorders, a combined eye-ear nose-throat item, the upper and lower gastrointestinal system, the hepatobiliary system, the kidney, genitourinary diseases, musculoskeletal diseases, endocrine/metabolic disorders, the neurological system, and behavioral-psychiatric disorders. Each single item was rated based upon the clinical data available according to the following algorithm: 1  =  no, 2  =  mild, 3  =  moderate, 4  =  severe, 5  =  life-threatening. No subjects obtained a score of 1 in our sample. After completion of the CIRS, two summary measures were constructed. First, the overall illness severity (SI) was represented by the mean of the 14 CIRS items (CIRS-SI). Second, the comorbidity index (CIRS-CI) was computed by counting the number of items for which moderate to severe pathology was reported (scores ≥3). As a result, the CIRS-CI can also be considered the number of clinically relevant concomitant diseases [17]–[21]. The total comorbidity index (CIRS-CI) ranged from 0 to 9. For analytical purposes, the CIRS-CI were then divided into tertiles obtaining three groups representing low (C1 = 0), medium (C2 = 1–2) and high (C3 ≥3) level of comorbidity.

### Calculations and statistical analyses

The observed data are normally distributed (Shapiro-Wilk W-Test) and presented as means ± Standard Deviation (SD). To assess differences among the two presented groups, an unpaired *t* test or a Pearson's Chi squared test were used, as appropriate. To evaluate the association of crash risk and comorbidity status, we divided the comorbidity and severity indices into tertiles. Numerical data indicating CIRS-CI were divided into three categories representing low (C1 = 0), medium (C2 = 1–2) and high (C3≥3) level. Ranks have been generated in ascending order and the mean rank of tied values was used for ties. The impact of comorbidity indices on crash risk was examined by logistic regression analyses and the odds ratio (OR) was presented to estimate the strength of the association.

Sample size calculation was estimated on an IBM PC computer by GPOWER software. The resulting total sample size, estimated according to a global effect size of 30% with type I error of 0.05 and a power of 99% was 238 patients. All p values presented are 2-tailed and a p≤0.05 was chosen for levels of significance. Statistical analyses were performed using SPSS 16 software package (SPSS, Inc., Chicago, IL).

## Results

### Population description and crash involvement

Table 1 shows demographic and clinical characteristics of the sample grouped by age range. All subjects (n = 562) had a mean age of 70 years with no difference in gender distribution. Stratifying population according age range, older subjects (age ≥70 years) had a significantly higher comorbidity and severity indices compared to younger subjects (age ≥70 years). The median age of the groups was 76 years (range  = 70–92 years) for the older group and 51 years (range  = 42–69 years) for younger group. Among all subjects studied, 172 (30.6%) had never driven in their lifetime, 63 (11.2%) had stopped driving, and 327 (58.2%) were current drivers. Among the older group (n = 355), only 210 subjects had a driving license, 151 currently drive, and 59 had stopped driving, while among 180 younger subjects with a driving license, (176) 97.7% currently drive (p = <.0001). Compared with younger drivers, current older drivers declared to drive few miles avoiding very long trips (p = .035). Older former drivers reported greater frequencies of cataract, poor vision, or mild cognitive impairment as well as a greater number of diseases, while all 4 former drivers among the younger group voluntarily stopped driving due to economic reasons (data not shown).

**Table 1:** Sample characteristics (n = 562).

	All (n = 562)	>70 years (n = 355)	<70 years (n = 207)	*p*
Age (years)	70.4±10.6	78.2±5.4	52.4±6.0	<.0001
Gender (M/F)	276/286	175/180	101/106	.454
CIRS-SI (score)	1.59±0.39	1.68±0.32	1.38±0.27	<.0001
CIRS-CI (score)	2.19±2.01	2.67±1.98	1.04±1.56	<.0001
Driving license n (%)	390 (69.3)	210 (59.1)	180 (86.9)	<.0001
Current Drivers n (%)	327 (58.1)	151 (42.5)	176 (85.0)	<.0001
Crashes n (%)	74 (13.1%)	41 (11.5%)	33 (15.9%)	.036

CIRS-SI =  Cumulative Illness Rating Scale-Severity Index; CIRS-CI =  Cumulative Illness Rating Scale-Comorbidity Index. Crashes number over the last 5 years. *p* = >70 years vs <70 years.

Older subjects had a significantly lower rate of crash involvement (p = .036) based on total crash numbers and lower percentage of current drivers then younger. Table 2 shows crash involvement rates per licensed drivers (n = 327) and the types of crashes in which the two age groups were injured over the last 5 years. The majority of crashes per current licensed drivers were found to be associated with the older group (χ^2^ = 3.935, p = .047) (Table 2). For both age groups, the majority of crashes were involved in a collision with another vehicle (75.6% of older group and 60.6% of younger group; χ^2^ = 8.108, p = .05). A greater proportion of younger drivers had collisions with fixed or other objects (χ^2^ = 8.308, p = .035); the most reported was a collision with a car mirror while driving. Older drivers mainly referred to collisions with a fixed object, during reversing or parking. For both age groups, the main maneuvers at the time of collision with another vehicle were driving straight ahead. No driver experienced multiple crashes in the last five years.

**Table 2:** Crash type by driver age group based on the number of current drivers (n = 327) over the last 5 years.

	>70 years (n = 151)	<70 years (n = 176)	*p*
	Count (%)	Count (%)	
Total crashes	41 (27.1)	33 (18.7)	.047
Collision with vehicle	31 (75.6)	20 (60.6)	.047
Collision with fixed or other object	10 (24.3)	13 (39.3)	.035

### Comorbidity score and crash involvement

**Figure 1:**
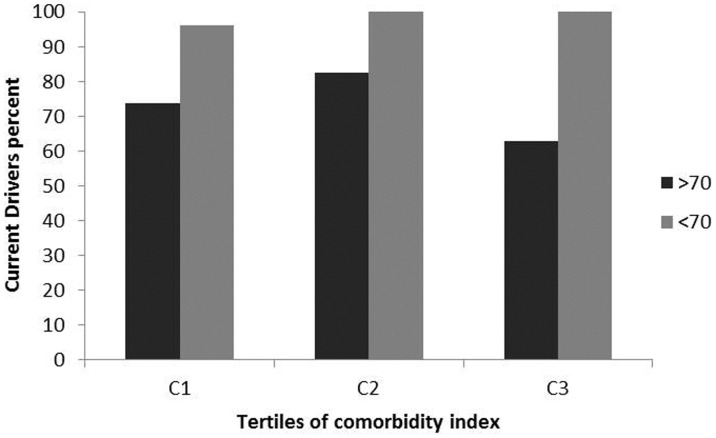
Percent of current drivers stratified by tertiles of comorbidity index among the two age groups. Drivers <70 (n = 176): C1 = 99 (96.1%), C2 = 50 (100%), C3 = 27 (100%); χ^2^ = 1.543, p = .462. Drivers >70 (n = 151): C1 =  28 (73.6%), C2 = 62 (82.6%), C3 = 61 (62.8%); χ^2^ =  7.301, p = .026.

**Figure 2:**
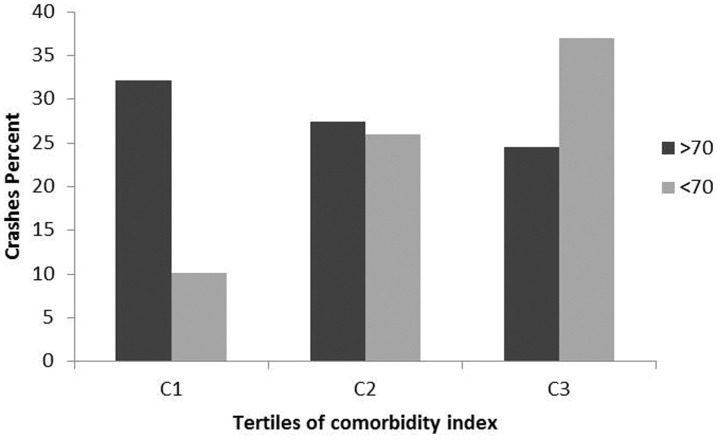
Crash percent stratified by tertiles of comorbidity index among the two age of current driver groups. Drivers <70 (n = 176): C1 = 99 (10.1%), C2 = 50 (26%), C3 = 27 (37%); χ^2^ = 5.885, p = .048. Drivers >70 (n = 151): C1 =  28 (32.1%), C2 = 62 (27.4%), C3 = 61 (24.5%); χ^2^ = 0.633, p = .729.

To better assess the effects of comorbidities on crash risk, we stratified all subjects by tertiles of comorbidity index, from lower to higher level (lower, C1 = 162; middle, C2 = 190; higher, C3 = 210). As expected in all populations with increasing age, comorbidity index significantly increased (C1: 63.5±11.3, C2: 69.9±9.6, C3: 75.4±7.9, p<0.0001). Stratifying only licensed drivers (n = 390) by comorbidity tertiles (C1 = 141; C2 = 125; C3 = 124), increasing level of comorbidity was associated with a reduced number of drivers (n = 127, n = 112, n = 88 respectively; χ^2^ = 16.206; p<.0001). Categorizing all subjects licensed to drive according to the range of age, the number of drivers among the younger population was significantly higher (χ^2^ = 18.219; p<.0001). Older drivers significantly decreased with increasing comorbidity index (p = .026) while no difference in the younger population was found (p = .462) (Figure 1). Considering only current licensed drivers, it was determined that with an increase in comorbidity index, the number of road accidents in the older population group decreased, while an opposite trend among younger drivers was found (p = .048) (Figure 2). Logistic regression models showed that in the younger population, the comorbidity index was significantly associated with crash involvement independent of gender and driving exposure (Table 3, model 1). A binomial regression analysis with logit link was constructed to assess a possible interaction between comorbidity and severity indices for crash risk (Table 3, model 2). This model showed a significant main effect for the comorbidity severity indices interaction for crash involvement risk. The same analyses conducted in the older population did not show any association between comorbidity index and crash involvement risk (B = −0.189, p = .517).

**Table 3:** Binary logistic regression analyses with crash involvement as dependent variable in younger drivers population study (n = 176).

	B	Odds Ratio	95% CI	*p*
**Model 1**				
Gender	0.610	1.841	0.566–5.982	.310
Driving exposure	0.153	1.166	0.585–2.324	.663
Comorbidity index	0.789	2.202	1.090–4.446	. 028
**Model 2**				
Gender	0.607	1.835	0.564–5.970	.313
Driving exposure	0.150	1.161	0.581–2.323	.672
Comorbidity index	0.745	2.105	0.701–6.324	.185
Severity index	0.151	1.163	0.539–4.116	.418
Comorbidity*Severity	0.728	2.117	1.048–4.185	.037

Comorbidity and severity indices expressed in tertiles.

Driving exposure expressed as low = 1, medium = 2, high  = 3 distance traveled.

## Discussion

This study aimed at investigating the direct relationship between comorbidity burden and vehicle crash involvement in a cohort of elderly and younger drivers. We found that comorbidity, measured by CIRS, is a self-restraint factor among older drivers and represents a predictor of vehicle crash involvement among the younger population, rather than in older people, independent of gender and driving exposure. Most younger subjects with a higher number of diseases continue to drive, while higher comorbidity level is associated with significantly less driving among the older group. Compared with previous studies [1], [2], our study shows that older subjects have a lower rate of crash involvement than younger drivers. When analysis was restricted to only current drivers, we found a significantly higher percentage of vehicle crash involvement among older compared to younger drivers.

The role of aging in crash risk is complex and dependent on multiple factors. As people age, deterioration of visual, cognitive, perceptual, and physical functions may increase their likelihood to be involved in traffic accidents [24]–[26]. Compared with younger drivers, older drivers have lower rates of crash involvements per capita, largely because older drivers are less likely to be involved in crashes since fewer older people have licenses and drive fewer miles compared to younger subjects [1], [2]. However, consistent with previous studies [1], [2], [27], after adjusting for the number of licensed drivers [27], [28], our study confirms that crash rates significantly increase in the older population. The reasons are still unclear and debated. As older drivers generally drive less distance per year than do drivers in other age groups, it has been hypothesized that the increased crash risk among older drivers is an artifact of the low mileage bias [29], [30]. Because older drivers typically drive less distance per trip and hence have lower accumulated driving distances per year, they have greater crash involvement per unit of distance compared to drivers with greater accumulated driving distances [30]. Accordingly when we asked about their driving pattern, all older drivers confirmed they drive fewer miles and avoid very long trips compared to the younger group.

Considering that numerous age-related diseases such as diabetes, poor vision, disability, and cognitive decline contribute to poor driving performance and crash involvement risk [5]–[10], [31], we asked whether the accumulation of diseases affecting many organs and tissues, expressed as comorbidity indices, may directly impact the higher crash rate. The effect of medications and comorbidities has been studied in crash and fatality data [32]; many diseases such as heart disease, stroke, and neurological conditions are crash predictors. Numerous studies identified comorbidities as predictors of older driver performance and driving pattern, while to the best of our knowledge, no study, so far, evaluated the direct impact of comorbidities on road crash risk among elderly drivers. Surprisingly, we found that licensed older drivers with higher level of comorbidity are less likely to drive, and thus are not involved in vehicle crashes. With increasing comorbidity index, we observed a significant lower number of former drivers among older subjects, while no difference among licensed younger drivers was found. This means that older subjects with high level of comorbidity are able to self-regulate driving. Moreover, comorbidities, not only do not impact driving pattern in the younger population, but also the number of crash involvement among younger current drivers significantly increases with increasing comorbidity level. Conversely, our data show a lessening trend among older current drivers in crash involvement, even if the difference did not reach statistical significance. Indeed, the logistic regression analyses show that comorbidity index is significantly associated with crash risk among younger subjects, while demonstrating no effect among older drivers. We found that the interaction between CIRS-IS and CIRS-CI is a significant predictor for crash involvement among younger driving populations. This finding emphasizes that comorbidity burden with increasing severity is a predictor for crash risk for younger individuals who even younger and sick still drive due to economic necessities (e.g., having to work or not having access to in-home social services compared with elderly subjects), while on contrary, represent a significant break among older subjects. Thus among younger drivers the number of diseases and their severity are predictor of crash involvement independent of gender and distance driven.

Older drivers are becoming a large group of road users, and will increase due to the aging population [1]. It is well established that older drivers are more likely to be injured in the event of a crash [3], [4], however our data show that older drivers are more prone to self limiting driving exposure with increasing comorbidities than their younger counterparts. These data highlight that older drivers should not be restricted from driving based upon their chronological age, but rather on their functional aging; thus older subjects with high level of comorbidity are able to self-regulate driving while comorbidity burden represents a significant risk factor for crash involvements among younger drivers.

Study strengths and limitations should be considered. Major strength is that, when assessing associations between comorbidity levels and crash involvement risk, the study design included control for other factors known to affect crash risk in drivers, such as driving exposure and gender. Another strength of this study is that it is based on a sample of 327 drivers, which enhanced the statistical power. Limitations include using self-reported crash involvement and driving exposure by memory, although there are strong evidences that these are valid estimates even in older subjects [22]. Another limitation of this study is that it is based on a secondary care based sample referred to our Department of Internal Medicine for diagnosis which reduces its generalizability. Indeed, considering that drugs belonging to neurological class are associated with higher crash risk among older subjects [33], we excluded all subjects taking antidepressant and sleep medications potentially affecting driving performance. However many other medications may affect safe driving and the lack of such information represents another potential limitation of the study. Thus further studies based on a general population sample or including medications as confounding variable are needed to generalize our findings.

In conclusion, our results have important practical implications and according with previous research suggest that drivers who overestimate their abilities are more likely to place themselves in situations that exceed their limitations [34], [35], such as higher crash involvements among younger drivers affected by multiple diseases. Our findings suggest that drivers with high comorbidity level need assessment independent of age while older subjects may be better at self regulating in this regard. Indeed in terms of policy implications, these findings strongly suggest that it is necessary to identify older drivers who are truly unsafe to drive and allow those who are safe to drive to keep driving as long as possible, maintaining their independence and quality of life.

## References

[1] Koppel S, Bohensky M, Langford J, Taranto D (2011) Older drivers, crashes and injuries. Traffic Inj Prev. 12(5): 459–67. 21972856 10.1080/15389588.2011.580802

[2] Cheung I, McCartt AT, Braitman KA (2008) Exploring the declines in older driver fatal crash involvement. Ann Adv Automot Med 52: 255–64. 19026242 PMC3256784

[3] Li G, Braver ER, Chen LH (2003) Fragility versus excessive crash involvement as determinants of high death rates per vehicle-mile of travel among older drivers. Accid Anal Prev 35: 227–235. 12504143 10.1016/s0001-4575(01)00107-5

[4] Lyman S, Ferguson SA, Braver E, Williams AF (2002) Older driver involvements in police reported crashes and fatal crashes: trends and projections. Inj Prev 8: 116–120. 12120829 10.1136/ip.8.2.116 PMC1730843

[5] Tervo T, Räty E, Sulander P, Holopainen JM, Jaakkola T, et al (2013) Sudden death at the wheel due to a disease attack. Traffic Inj Prev 14(2): 138–44. 23343022 10.1080/15389588.2012.695827

[6] Uc EY, Rizzo M (2008) Driving and neurodegenerative diseases. Curr Neurol Neurosci Rep 8(5): 377–83. 18713573 10.1007/s11910-008-0059-1 PMC3097428

[7] Dubinsky RM, Stein AC, Lyons K (2000) Practice parameter: risk of driving and Alzheimer's disease (an evidence-based review): report of the quality standards subcommittee of the American Academy of Neurology. Neurology 54(12): 2205–11. 10881240 10.1212/wnl.54.12.2205

[8] Decina LE, Staplin L (1993) Retrospective evaluation of alternative vision screening criteria for older and younger drivers. Accid Anal Prev 25: 267–75. 8323661 10.1016/0001-4575(93)90021-n

[9] Owsley C, McGwin G Jr, Searcey K (2013) A population-based examination of the visual and ophthalmological characteristics of licensed drivers aged 70 and older. J Gerontol A Biol Sci Med Sci 68(5): 567–73. 22982690 10.1093/gerona/gls185 PMC3623480

[10] Popescu ML, Boisjoly H, Schmaltz H, Kergoat MJ, Rousseau J, et al (2011) Age-related eye disease and mobility limitations in older adults. Invest Ophthalmol Vis Sci 52(10): 7168–74. 21862652 10.1167/iovs.11-7564

[11] Sims RV, Ahmed A, Sawyer P, Allman RM (2007) Self-reported health and driving cessation in community-dwelling older drivers. J Gerontol A Biol Sci Med Sci. 62(7): 789–93. 17634328 10.1093/gerona/62.7.789

[12] Choi M, Mezuk B, Rebok GW (2012) Voluntary and involuntary driving cessation in later life. J Gerontol Soc Work 55(4): 367–76. 22574868 10.1080/01634372.2011.642473

[13] Forrest KY, Bunker CH, Songer TJ, Coben JH, Cauley JA (1997) Driving patterns and medical conditions in older women. J Am Geriatr Soc. 1997 45(10): 1214–8. 10.1111/j.1532-5415.1997.tb03772.x 9329483

[14] Classen S, Horgas A, Awadzi K, Messinger-Rapport B, Shechtman O, et al (2008) Clinical predictors of older driver performance on a standardized road test. Traffic Inj Prev. 9(5): 456–62. 18836957 10.1080/15389580802260026

[15] Parmelee PA, Thuras PD, Katz IR, Lawton MP (1995) Validation of the Cumulative Illness Rating Scale in a geriatric residential population. J Am Geriatr Soc 43: 130–7. 7836636 10.1111/j.1532-5415.1995.tb06377.x

[16] Conwell Y, Forbes NT, Cox C, Caine ED (1993) Validation of a measure of physical illness burden at autopsy: the Cumulative Illness Rating Scale. J Am Geriatr Soc 41: 38–41. 8418120 10.1111/j.1532-5415.1993.tb05945.x

[17] Nagaratnam N, Gayagay G Jr (2007) Validation of the Cumulative Illness Rating Scale (CIRS) in hospitalized nonagenarians. Arch Gerontol Geriatr 44: 29–36. 16621072 10.1016/j.archger.2006.02.002

[18] Boulos DL, Groome PA, Brundage MD, Siemens DR, Mackillop WJ, et al (2006) Predictive validity of five comorbidity indices in prostate carcinoma patients treated with curative intent. Cancer 106: 1804–14. 16534794 10.1002/cncr.21813

[19] Giaquinto S, Palma E, Maiolo I, Piro MT, Roncacci S, et al (2001) Importance and evaluation of comorbidity in rehabilitation. Disabil Rehabil 23: 296–9. 11354582 10.1080/096382801750143643

[20] Bo M, Cacello E, Chiggia F, Corsinovi L, Bosco F (2007) Predictive factors of clinical outcome in older surgical patients.Arch Gerontol Geriatr. 44: 215–24. 10.1016/j.archger.2006.05.007 16870278

[21] Extermann M (2000) Measuring comorbidity in older cancer patients. Eur J Cancer 36: 453–71. 10717521 10.1016/s0959-8049(99)00319-6

[22] McGwin G Jr, Owsley C, Ball K (1998) Identifying crash involvement among older drivers: agreement between self-report and state records Accid Anal Prev. 30(6): 781–91. 10.1016/s0001-4575(98)00031-1 9805521

[23] Murakami E, Wagner DP (1997) Comparison between computer assisted self-interviewing using GPS with retrospective trip reporting using telephone interviews. Washington, DC: Federal Highway Administration, US Department of Transportation,

[24] Dewar RE (2002) Age differences – drivers old and young. In: Dewar, R.E., Olson, P., (Eds.), Human Factors in Traffic Safety. Lawyers & Judges Publishing, Tucson, AZ 209–233.

[25] Sims RV, McGwin G Jr, Pulley LV, Roseman JM (2001) Mobility impairments in crash-involved older drivers. J. Aging Health 13 (3): : 430–438. 10.1177/089826430101300306 11813734

[26] Cheung I, McCartt AT (2011) Declines in fatal crashes of older drivers: changes in crash risk and survivability. Accid Anal Prev 43(3): 666–74. 21376853 10.1016/j.aap.2010.10.010

[27] Holland CA (2002) Older Drivers: A Literature Review. Road Safety Research Report No. 25, Department for Transport.

[28] Cerrelli EC (1995) Crash Data and Rates for Age-sex Groups of Drivers. National Center for Statistics and Analyses, National Highway Administration, Washington, DC.

[29] Langford J, Koppel S, McCarthy D, Srinivasan S (2008) In defence of the lowmileage bias. Accid. Anal. Prev. 40 (6): : 1996–1999. 10.1016/j.aap.2008.08.027 19068306

[30] Langford J, Methorst R, Hakamies-Blomqvist L (2006) Older drivers do not have a high crash risk – a replication of low mileage bias. Accid. Anal. Prev. 38 (3): : 574–578. 10.1016/j.aap.2005.12.002 16426560

[31] McGwin G Jr, Sims RV, Pulley L, Roseman JM (1999) Diabetes and automobile crashes in the elderly. A population-based case-control study. Diabetes Care. 22(2): 220–7. 10333937 10.2337/diacare.22.2.220

[32] McGwin G Jr, Sims RV, Pulley L, Roseman JM (2000) Relations among chronic medical conditions, medications, and automobile crashes in the elderly: a population-based case-control study. Am J Epidemiol. 152(5): 424–31. 10981455 10.1093/aje/152.5.424

[33] Hemmelgarn B, Suissa S, Huang A, Boivin JF, Pinard G (1197) Benzodiazepine use and the risk of motor vehicle crash in the elderly. JAMA 278(1): : 27–31. 9207334

[34] MacDonald L, Myers AM, Blanchard RA (2008) Correspondence among older drivers' perceptions, abilities, and behaviors. Topics in Geriatric Rehabilitation 24: 239–252.

[35] Marottoli RA, Richardson ED (1998) Confidence in, and self rating of driving ability among older drivers. Accident Analysis and Prevention 30: 331–336. 9663292 10.1016/s0001-4575(97)00100-0

